# Pattern of un-operated Grown Up Congenital Heart (GUCH) patients presenting to a Tertiary Care Cardiac Institute of Punjab

**DOI:** 10.12669/pjms.35.4.878

**Published:** 2019

**Authors:** Abdul Razzaq Mughal, Rubina Tousif, Asif Rashid Alamgir, Anjum Jalal

**Affiliations:** 1 Abdul Razzaq Mughal FCPS (Paed Med), FCPS (Paed Card), Department of Pediatric Cardiology, Faisalabad Institute of Cardiology, Faisalabad, Pakistan; 2 Rubina Tousif FCPS (Paeds Med), Department of Cardiology, Children Hospital Faisalabad, Pakistan; 3 Asif Rashid Alamgir (MS Anesthesia), Department of Cardiac Anesthesia, Faisalabad Institute of Cardiology, Faisalabad, Pakistan; 4 Anjum Jalal FRCS, FCPS-CS, FRCS-CTh, Department of Cardiac Surgery, Faisalabad Institute of Cardiology, Faisalabad, Pakistan

**Keywords:** Congenital Heart Disease, Grown-up congenital heart disease, Morbidity

## Abstract

**Objectives:**

To identify the pattern of un-operated grown up congenital heart defects at our tertiary care cardiac institute.

**Methods:**

This is a prospective observational study. All un-operated GUCH patients who presented to Faisalabad Institute of Cardiology (FIC) from May 2017 to 30th July 2017 were enrolled. Diagnosis was established on Transthoracic Echocardiography done by dedicated pediatric cardiologist at FIC. The mode of presentation, presenting complaints, type, severity, complications and co-morbid conditions of CHD were recorded.

**Results:**

A total of 200 consecutive patients were enrolled. Mean age was 29.92 ± 11.21 years. There were 104 females (52%) and 96 males (48%). Majority of patients presented in Out-Patient Department (84%) while 16% presented in emergency (n=32). The most common cardiac anomalies were: Atrial Septal Defect (ASD) 41.5% (83), Tetralogy of Fallots (TOF) 42 (21%), Ventricular Septal Defect (VSD) 28 (14%) and Patent ductus arteriosus (PDA) 8 % (16). Cyanotic CHD was present in 43% (86) while TOF was the most common of it. The disease was of moderate complexity in 77.5% patients. Certain complications like Pulmonary hypertension 69(34.5%), Eisenmenger 33(16.5%), Rhythm disturbances 15 (7.5%), Infective endocarditis 5(2.5%) were also present along with co-morbid conditions like coronary artery disease (1.5% and systemic hypertension (2.5%). Dyspnea on exertion (59.5%) followed by cyanosis (41%) were the most common presenting complaints. The most common reason for hospital admission was cardiac signs and symptoms (19.5%) followed by cardiac catheterization (10.5%).

**Conclusion:**

The ASD, TOF, VSD and PDA remain the most common CHD in descending order while pulmonary hypertension, Eisenmenger, heart failure, arrhythmias, infective endocarditis and stroke were the common complications of CHD at this particular age.

## INTRODUCTION

The improved awareness and management of congenital heart defects (CHD) has resulted in increased number of grown up congenital heart disease (GUCH) patients seeking treatment. Over 80% of the babies with CHD today will survive into adulthood.^1^ Currently overall prevalence of CHD in the adult population is about 3000 per million.^2^ There is a lack of data on the spectrum of GUCH patients in Pakistan.^3^ There are two different categories of GUCH patients; one who previously have been undiagnosed; and the others who have been diagnosed, treated, in their childhood and thereafter require follow-up during their adult years.

The types of GUCH diseases have been classified as complex, moderate, and mild lesions.^4^ Most of the GUCH patients usually have simple cardiac malformations; a few have complex diseases, and some have survived at the cost of secondary pulmonary hypertension. Most of the patients having complex congenital heart defects die at early age if not treated in time. Few of defects like atrial septal defect (ASD), coarctation of the aorta (CoA), Ebstein’s anomaly, and congenitally corrected transposition of the great arteries (cCTGA) may be diagnosed for the first time in adult life. Pakistan is a developing country and late presentation of patients with CHD is due to non-availability of services as well as economic and cultural restraints. There are very few specialized pediatric cardiology centers in Pakistan which offer treatment of CHD.^5^ The aim of this study was to find out the pattern and the frequency of the various forms of un-operated grown up congenital heart disease in our set up. This would help in training the man power accordingly while expanding the services.

## METHODS

It was a descriptive prospective study conducted in Pediatric cardiology department of Faisalabad Institute of cardiology (FIC) Faisalabad, Pakistan. The FIC is the largest tertiary care cardiac center of Faisalabad with a total bed strength of 250. The study was carried out for three months from May 2017 to 30th July 2017. Approval of hospital’s ethics committee was obtained and there was no conflict of interest. A total of two hundred consecutive patients of 18 years or above with a diagnosis of structural congenital heart defect were included in the study. They underwent echocardiography by dedicated pediatric cardiologist at FIC. The patients who underwent surgery or interventional treatment for CHD were excluded. Moreover, the patients suffering from bicuspid aortic valve or congenital electrophysiological problems were also excluded.

The CHD was defined as a structural abnormality of the heart or intra-thoracic great vessels that has actual or potential functional significance. The demographic profile, mode of presentation and reason for admission were noted. Presenting symptoms compelling the patient to stay in the hospital were also noted including dyspnea on exertion, cyanosis, chest pain, edema, fever, palpitation, hemoptysis and syncope. Functional class of the patient according to New York Heart Association was assessed. All patients underwent detailed echocardiography by the same consultant pediatric cardiologist (Author ARM). The diagnosis of the structural heart abnormalities was classified according to the following criteria given in Table-I.

**Table I:** Classification of congenital heart diseases in adult patients based upon severity of complexity.

Mild
^Isolated congenital aortic valve disease, Isolated congenital aortic valve disease, Isolated congenital mitral valve disease (e.g., except parachute valve, cleft leaflet) Isolated patent foramen ovale. Small atrial septal defect. Isolated small ventricular septal defect (VSD) (no associated lesions). Mild pulmonic stenosis (PS)^

**^Moderate^**

Aorto-left ventricular fistulae. Anomalous pulmonary venous drainage. Atrioventricular canal defect (Partial or Complete). Coarctation of the aorta. Ebstein’s anomaly. Infundibular right ventricular outflow obstruction. Patent ductus arteriosus (not closed). Pulmonary valve regurgitation (moderate to severe). Pulmonary valve stenosis (Moderate to severe). Sinus of Valsalva fistula/aneurysm. Sinus venosus atrial septal defect. Ostium Primum ASD. Sub-valvular or supra-valvular aortic stenosis (except HOCM). Tetralogy of Fallot. Ventricular septal defect (VSD) with Absent valve or valves. VSD with aortic regurgitation. VSD with Coarctation of the aorta. VSD with right ventricular outflow tract obstruction. VSD with Straddling tricuspid/mitral valve. VSD with Sub-aortic stenosis

**Severe**

^Cyanotic congenital heart (all forms), Double-outlet ventricle, Eisenmenger syndrome, Mitral Atresia, Single ventricle (also called double inlet or outlet, common or primitive, Pulmonary atresia (all forms), Transposition of the great arteries, Tricuspid Atresia, Truncus arteriosus/hemitruncus, Abnormalities of atrioventricular or ventriculoarterial connection not included above (i.e., crisscross heart, isomerism, heterotaxy syndromes, ventricular inversion), Pulmonary vascular obstructive disease^

*Ref:* Webb DG, Roberta G. Care of the Adult with Congenital Heart Disease, October 2-3, 2000: 32nd Bethesda Conference. JACC. 2001;37(5):1161-1198.

The leading or main diagnosis was chosen if there were more than one structural abnormalities. The patients were categorized as cyanotic or acyanotic on the basis of echocardiography and presence or absence of cyanosis. Any patient of CHD having less than 92 % oxygen saturation or clinically visible cyanosis was labeled as cyanotic CHD. Patients of severe pulmonary hypertension having clearly mentioned right to left shunt on echocardiography or catheter based calculated pulmonary vascular resistance (PVR) more than 8 Wood Units were labeled as Eisenmenger.

The patients and/or parents of the patients were interviewed by the first author in a comfortable environment to get all the required information after taking informed consent and assuring confidentiality of the data. The data of patients was entered in the dedicated database of the hospital (Cascade Database, Lahore). The means and the standard deviations were calculated for the quantitative variables and the frequencies and percentages were calculated for qualitative variables. Post stratification Chi-Square test was applied and P-value ≤ 0.05 was considered as significant.

## RESULTS

A total of 200 consecutive patients who fulfilled the inclusion and exclusion criteria of the study were enrolled during the study period. Their ages ranged from 18 to 60 years with mean age of 29.92+11.21 years. Majority of patients were between 18 to 45 years of age (88.5%, n=177) while 11.5% were above 45 years of age (n=23). There were 52% female patients and 48% male patients. Most of the subjects were from rural areas of the Punjab province (52%, n=104), 95 patients (47.5%) from Faisalabad and other urban areas of Punjab while one patient (0.5%) belonged to urban area of Sindh province. Quite significant number of patients were uneducated (43%, n=86) while 57% of patients were having primary level education or above.

Majority of patients (84%, n=168) presented in outpatient department (OPD) of the hospital while 16% patients (n=32) presented in emergency due to severity of symptoms. Seventy-one patients were admitted due to different reasons including severe symptoms (n=39, 19.5%), cardiac catheterization (n=18, 9%), cardiac intervention (n=3, 1.5 %) and cardiac surgery (11, 5.5%). Dyspnea on exertion was the most common presenting symptom (n=119) followed by cyanosis (n=41). On presentation the New York Heart Association (NYHA) functional class was assessed and most of the patients were in NYHA functional class II (n=106, 53%). Sixty patients (30%) were in NYHA functional class III, 29 patients were in functional class I (14.5%) while only 5 patients were in functional class IV (2.5%).

The types of cardiac abnormalities detected in the patients are shown in Table-II. Majority of the patients were of acyanotic CHD (n=114, 57%) while 86 (43%) patients had cyanotic heart disease including Eisenmenger disease. The most common cyanotic congenital heart disease was TOF (n=42, 21%) followed by Eisenmenger syndrome which was 16.5% (n=33). As regards complexity of disease 77.5% patients were of moderate complexity (n=155), 21 % were of severe complexity (n=42) while only 1.5% (n=3) were of mild complexity. A significant number of complications and associated problems were noted in this population of un-operated GUCH patients. The commonest complication was pulmonary hypertension with or without Eisenmenger physiology. The complications and their respective frequencies are mentioned in Table-III.

**Table II:** Anatomical types of cardiac defects in GUCH population with gender distribution.

S. No.	Anatomical Diagnosis	Gender		Frequency	Percentage of Total

		Male n=96	Female n=104		
1.	Secundum ASD	28	44	72	36
2	Partial AVSD	2	3	5	2.5
3	Sinus Venosus ASD	1	5	6	3
4	Complete AVSD	1	4	5	2.5
5	TOF	27	15	42	21
6	VSD	19	9	28	14
7	PDA	2	14	16	8
8	Valvular PS	2	4	6	3
9	LVOTO	0	1	1	0.5
10	RVOTO	2	0	2	1
11	Ebstein Anomaly	2	0	2	1
12	DORV	2	0	2	1
13	L-TGA	3	0	3	1.5
14	TGA	0	1	1	0.5
15	Pulmonary atresia	1	2	3	1.5
16	Univentricular Heart	2	1	3	1.5
17	RSOV	2	1	3	1.5

*Abbreviations:* Atrial Septal Defect (ASD); Atrio-ventricular Septal Defect AVSD; Tertalogy of Fallot (TOF); Ventricular Septal Defect (VSD); Patent Ductus Arteriosus (PDA); Pulmonary Stenosis (PS); Left Ventricular Outflow Tract Obstruction (LVOTO); Right Ventricular Outflow Tract Obstruction (RVOTO); Double Outlet Right Ventricle (DORV); Transposition of Great Arteries (TGA); Ruptured Sinus of Valsalva (RSOV)

**Table III:** Frequency of complications in GUCH patients.

S. No.	Complications & Associated Problems in GUCHD Patients	Frequency	Percentages
1	Pulmonary Hypertension (PH)	69	34.5
	Mild PH	2	1
	Moderate PH	10	5
	Severe PH	57	28.5
2	Eisenmenger	33	16.5
3	Coronary artery disease (CAD)	3	1.5
	Left main stem disease	2	1
	Two vessels CAD	1	0.5
4	Rhythm abnormalities	15	7.5
	Atrial Fibrillation	4	2
	Atrial Flutter	5	2.5
	Supra ventricular Tacchycardia	4	2
	Complete Heart Block	2	1
5	Endocarditis	5	2.5
	Vegetation Positive	3	1.5
	Culture Positive	2	1
6	Systemic Hypertension	5	2.5
7	Neurological Problems	2	1
	Brain Abscess	1	0.5
	Hemiplegia	1	0.5

## DISCUSSION

The data of adult congenital heart defects is mostly available in literature up to a maximum age of 40 years. In our study mean age of patients at presentation was 29.92 years while majority were below 45 years (88.5%). This is comparable with Gatzoulis et al.^6^ who reported mean age of 31.7 years in patients attending adult clinic for CHD. There is no significant gender based difference among GUCH population presenting in our study.

According to the published data on GUCH population simple cardiac defects are more common as compared to complex one. The ASD is the most common CHD in our study (41.5%) which is comparable with Giannoglou et al.^7^ who reported that ASD was present in 43.3% of adult patients who underwent cardiac catheterization.

The incidence of un-operated Tetralogy of Fallots (TOF) in adults is variable in different parts of the world. Ahmad et al.^8^ reported the incidence of TOF as 7.14%. Similarly, in study of Wu et al.^9^ the incidence of TOF was 15.53%. The TOF was the second most common defect (21%) in our study and number one in cyanotic congenital heart defects. The reason for high incidence of TOF in our study could be a natural selection of such cases. It is well established that ventricular septal defect is the most common CHD in children but these patients usually get operated or die of complications before reaching adulthood. Hence, VSD are not as common in GUCH than in younger patients. Ventricular septal defect was the 3rd most common defect in our GUCH population (14%) which is consistent with an epidemiological study conducted in India by Bhardwaj et al.^10^ where incidence of VSD was 14%. This clearly shows that the countries of this particular region have almost similar lack of facilities for heart surgery of new born and infants children. The incidence of PDA in GUCH population shows a wide range from 7.7% to 18.5% reported in the literature across the world.^3^,^8^,^11^ In our study the incidence was 8%. This shows consistency in frequency in various parts of country as the figures in another recent report from Pakistan showed 8.2%.^3^

The decreasing incidence of PDA in adult population is due to easily available treatment of PDA in many centers. Atrioventricular septal defects (AVSD) are very uncommon defects in GUCH population. In a study conducted in different hospitals of Iran^12^ there were only 12 patients of AV canal defect (0.98%) detected in 9-years. In our study AVSD patients were only 5% including partial as well as complete AVSD (2.5% each). Valvular Pulmonary stenosis (PS) cases were only 3% in our study which is quite less than the Dutch CONCOR national registry for adult congenital heart disease^13^ in which 7% of patients were having valvular PS. Rest of the grown up congenital heart defects in our study were of less than 2% frequency. These included Pulmonary atresia, uni-ventricular heart, L-TGA and RSOV, RVOTO, Ebstein anomaly, TGA and DORV. The extremely lower incidence of such disease in our country is due to the severity of such defects leading to early death.

There were 57% patients with acyanotic CHD while 43% were of cyanotic CHD. Neidenbach et al.^14^ studied 821 patients of GUCH for non-cardiac co-morbidities where 91.6% of patients were of acyanotic CHD and 8.4% were of cyanotic CHD. In our study the incidence of cyanotic CHD patients is high where at the top of the list is TOF (21%) followed by cases of Eisenmenger physiology (16.5%). Rest of cyanotic CHD are less common.

In different parts of the world there is variation in the complexity of GUCH. In a study conducted in Greece by Giannakoulas et al.^15^ 47% of patients had mildly complex CHD, 37% had moderate while 15% had severe complexity. Similarly Helm et al.^16^ did a nationwide survey in Germany where 21.8% of patients were of simple complexity (n=398), 33.2% of moderate complexity (n =606), 38.2% of severe Complexity (n =699) while 6.8% were of unclassified CHD. In our study majority of patients (77.5%) were of moderate complexity (155) while 21 % of patients were of severe complexity and only 1.5% of mild complexity. The plausible reason for very low number of mild complexity patients is non-presentation due to mild symptoms which are generally ignored in our predominantly poor and illiterate population. Contrarily the patients with severe complexity are lesser in number, because of death at early age.

Dyspnea on exertion is the common mode of presentation in GUCH population. Tchoumi^17^ in his study reported 44 % of patients having NYHA class III and 7% having class IV. The common presenting symptom in our study is not different from other part of the world and 59.5 % of our patients presented with complaint of DOE while 53% of patients were in NYHA class II and 30% were in NYHA class III.

Pulmonary hypertension is a dreadful complication of un-operated CHD. In study conducted by Favilli et al.^18^ the prevalence of Eisenmenger was 1.2% while that of PH was 6%. Similarly, Duffels et al.^19^ reported 4.2% prevalence of PH. In our study 57 patients had severe PH while 16.5 % cases proved to be of Eisenmenger physiology. Our study clearly shows that patients of GUCH in this particular region of the world already have developed complications like PH and Eisenmenger before they present for treatment.

The arrhythmias are the most common cardiac reason for admission to the hospital, and atrial flutter is the most frequent disorder of rhythm, usually related to hemodynamic disturbances^20^ According to a study done by Bouchardy et al.^21^, 15% of adults with congenital heart disease develop atrial arrhythmias which is quite high as compared to our study where 6.5 % of patients had non valvular supra ventricular arrhythmias. Warnes CA^22^ in his study described cCTGA as the most common CHD associated with conduction disorders and 50 % of these patients develop complete heart block spontaneously till age 50 years. The complete heart block (CHB) incidence was only 1% in our study but it was 50% in subgroup of cCTGA.

Infective endocarditis is a known complication in un-operated GUCH patients. Verheugt et al.^23^ analyzed registry data of 10210 patients found that main predictors of infective endocarditis in GUCH patients included gender, multiple heart defects and previous history of infective endocarditis, supraventricular tachycardia and cerebrovascular accidents in childhood.^23^ In a study conducted by Ali SKM et al.^24^ found 1.54% of GUCH patients had endocarditis. In our study five patients (2.5%) had endocarditis which shows a higher incidence in this part of the world.

Children and adults with CHD are at increased risk of stroke due to secondary erythrocytosis, paradoxical embolism, and other mechanisms.^25^ In our study Neurological issues were present in only two patients. One of them had TOF and developed brain abscess and the other had Eisenmenger syndrome and developed massive brain infarction.

### Limitations of this study

It is based on a sample from a tertiary care cardiac center and may not truly reflect the exact situation in the community at large. Moreover, a snapshot of three months only represents the cases seen during that short duration. In Pakistan, there is no organized referral system for patients hence the presentation of patients to a particular center is generally determined by the impression of population at large about the type of services available at that particular center.

## CONCLUSION

The most common cardiac defect in GUCH population in our society is ASD. The patients presenting with Eisenmenger syndrome is reaching a very worrisome and alarming proportion (16.5%). This highlights the need for expansion of pediatric cardiology services in the country on priority.

### Author`s Contribution

**ARM:** Conceived, designed and did statistical analysis & editing of manuscript.

**RT and ARA:** Helped in data collection and manuscript review.

**AJ:** Supervised the study and did final review, editing and approval of manuscript

## References

[1] Congenital Heart disease statistics (2003). British Heart Foundation Health Promotion Research Group, Department of Public Health, University of Oxford.

[2] Mulder BJ (2012). Epidemiology of adult congenital heart disease:Demographic variations worldwide. Neth Heart J.

[3] Shah MUA, Jalal A, Rasheed I, Siddiqui MMA, Majeed S, Kayani AM (2015). Surgery of Grown up Congenital Hearts (GUCH) in Pakistan;What is different from the developed countries?. Pak J Med Health Sci.

[4] Webb DG, Roberta G (2001). Care of the Adult with Congenital Heart Disease, October 2-3 2000: 32nd Bethesda Conference. J Amer Coll Card.

[5] Mughal AR, Sadiq M, Hyder SN, Qureshi AU, AShah SS, Khan MA (2011). Socioeconomic status and impact of treatment on families of children with congenital heart disease. J Coll Physicians Surg Pak.

[6] Gatzoulis MA, Hechter S, Siu SC, Webb GD (1999). Outpatient clinics for adults with congenital heart disease:increasing workload and evolving patterns of referral. Heart.

[7] Giannoglo GD, Antoniadis AP, Chatzizisis YS, Louridas GE (2009). Adult Congenital Heart Disease Investigated with Cardiac Catheterization Over A 20-Year Period. Open Cardiovasc Med J.

[8] Ahmad F, Adnan Y, Faheem M, Noor L, Shah ST, Ullah I (2012). Spectrum of congenital heart diseases (CHD) in older children and adult population. Pak Heart J.

[9] Wu MH, Lu CW, Chen HC, Kao FY, Huang SK (2018). Adult Congenital Heart Disease in a Nationwide Population 2000-2014: Epidemiological Trends, Arrhythmia, and Standardized Mortality Ratio. J Am Heart Assoc.

[10] Bhardwaj R, Rai SK, Yadav AK, Lakhotia S, Agrawal D, Kumar A, Mohapatra B (2015). Epidemiology of Congenital Heart Disease in India. Congenit Heart Dis.

[11] Jassim AR, Alhadad HS, Nassrullah HAA (2017). Clinical and echocardiographic patterns of congenital heart diseases in adults in Karbala Province, Iraq. Iraq Med J.

[12] Rahim F, Ebadi A, Saki G, Remazani A (2008). Prevalence of Congenital Heart Disease in Iran:A Clinical Study. J Med Sci.

[13] Verheught CL, Uiterwaal CS, Van der Velde ET, Meijboom FJ, Pieper PG, Van Dijk AP (2010). Mortality in adult congenital heart disease. Eur Heart J.

[14] Neidenbach RC, Lummert E, Vigl M, Zachoval R, Fischereder M, Engelhardt A (2018). Non-cardiac co-morbidities in adults with inherited and congenital heart disease:report from a single center experience of more than 800 consecutive patients. Cardiovasc Diagn Ther.

[15] Giannakoulas G, Vasiliadis K, Frogoudaki A, Ntellos C, Tzifa A, Brili S (2017). Adult congenital heart disease in Greece:Preliminary data from the CHALLENGE registry. Int J Cardiol.

[16] Helm PC, Kaemmerer H, Breithardt G, Sticker EJ, Keuchen R, Neidenbach R (2017). Transition in Patients with Congenital Heart Disease in Germany:Results of a Nationwide Patient Survey. Front Pediatr.

[17] Tchoumi JCT, Butera G (2013). Profile of cardiac disease in Cameroon and impact on health care services. Cardiovasc Diagn Ther.

[18] Favilli S, Santoro G, Ballo P, Arcangeli C, Bovenzi FM, Chiappa E (2012). Prevalence and clinical characteristics of adult patients with congenital heart disease in Tuscany. J Cardiovasc Med.

[19] Duffels MGJ, Engelfriet PM, Berger RMF, van Loon RLE, Hoendermis E, Vriend JWJ (2007). Pulmonary arterial hypertension in congenital heart disease:An epidemiologic perspective from a Dutch registry. Int J Cardiol.

[20] (2002). Grown-up congenital heart (GUCH) disease:current needs and provision of service for adolescents and adults with congenital heart disease in the UK. Report of the British Cardiac Society Working Party. Heart.

[21] Bouchardy J, Therrien J, Pilote L, Ionescu-Ittu R, Martucci G, Bottega N (2009). Atrial Arrhythmias in Adults with Congenital Heart Disease. Circulation.

[22] Warnes CA (2006). Transposition of great arteries. Circulation.

[23] Verheugt CL, Uiterwaal CS, van der Velde ET, Meijboom FJ, Pieper PG, Veen G (2011). Turning 18 with congenital heart disease:prediction of infective endocarditis based on a large population. Eur Heart J.

[24] Ali SKM, Elmahdi LM, Ibrahim M (2010). Diagnosis and management of adults with congenital heart disease:the experience of the Sudan Heart Centre. Sudan Med J.

[25] Hoffmann A, Chockalingam P, Balint OH, Dadashev A, Dimopoulos K, Engel R (2010). Cerebrovascular accidents in adult patients with congenital heart disease. Heart.

